# Beyond Resilience: Physician Overtime as an Ethical Challenge for Sustainable Healthcare

**DOI:** 10.7759/cureus.114740

**Published:** 2026-08-18

**Authors:** Marta Dias, Patrícia P Almeida

**Affiliations:** 1 Occupational Medicine, Coimbra University Hospitals, Coimbra, PRT; 2 Occupational Medicine, Médio Tejo Hospital Centre, Torres Novas, PRT

**Keywords:** bioethics, burnout, physician overtime, physician well-being, sustainable healthcare

## Abstract

Overtime work, defined as recurrent work performed beyond regular contractual hours across clinical settings, has become an increasingly normalized feature of modern healthcare systems, often viewed as a marker of professional dedication rather than a sign of structural imbalance. However, excessive working hours contribute to physician burnout, impaired well-being, and potential risks to patient safety. From a bioethical perspective, sustainable healthcare requires protecting both patients and the professionals who provide care. Addressing physician overtime demands a shift from a culture of individual endurance toward organizational responsibility, supported by workforce planning, effective workload management, and enforcement of working-hour policies, while promoting working environments that safeguard dignity, safety, and long-term healthcare resilience.

## Editorial

Overtime work was historically intended to address extraordinary clinical demands. Yet in many healthcare systems it has gradually evolved into a structural expectation rather than an exception. This transformation raises important ethical questions regarding the sustainability of healthcare models that depend on continuous professional overextension.

Medicine has historically valued dedication, responsibility, and commitment to patients. While these values remain fundamental to clinical practice, they may also contribute to a professional culture in which self-sacrifice and endurance are perceived as indicators of competence. From medical training onward, physicians are frequently exposed to environments where accepting excessive workloads is normalized, and establishing boundaries may be interpreted as a lack of commitment. This culture risks transforming systemic challenges, such as workforce shortages and organizational inefficiencies, into individual responsibilities carried by physicians [1,2].

Physician overtime refers broadly to work performed beyond regular contractual hours, including prolonged shifts, additional clinical duties, or excessive on-call commitments when these become a recurrent rather than exceptional feature of practice. Although working-hour arrangements vary across healthcare systems and medical specialties, the ethical concerns discussed are broadly applicable whenever excessive working hours become normalized.

The consequences of persistent overtime extend beyond personal fatigue. Chronic exposure to excessive work demands has been strongly associated with burnout, emotional exhaustion, reduced professional satisfaction, sleep disruption, and adverse effects on physical and psychological health [1-3]. These outcomes should not be viewed merely as individual difficulties but as occupational health concerns affecting the functioning and resilience of healthcare systems.

Physician well-being is also directly connected to patient safety. Fatigue, emotional exhaustion, and psychological distress may compromise attention, decision-making, communication, and empathy, increasing vulnerability to clinical errors and reducing the quality of care delivered [4]. Therefore, protecting physicians from chronic overwork represents not only an occupational responsibility but also a patient safety priority.

The normalization of excessive working hours reflects broader structural weaknesses within healthcare organizations. Systems that depend on prolonged availability and unpaid or excessive overtime may temporarily maintain service delivery but risk creating long-term consequences, including dissatisfaction, workforce turnover, and worsening shortages. Sustainable healthcare cannot rely indefinitely on the personal sacrifice of professionals [3,5].

Physicians often experience an ethical conflict between their professional obligation to patients and their obligation to preserve their own health. Healthcare organizations may inadvertently rely on this sense of duty, transforming professional commitment into a mechanism that compensates for structural deficiencies. This creates a moral paradox in which acting altruistically may ultimately undermine both physician well-being and patient safety.

The ethical implications of physician overtime extend beyond excessive working hours alone. Healthcare systems must balance physicians' professional obligation to provide timely and continuous patient care with their right to safe working conditions and adequate rest. Although emergency demands, workforce shortages, and limited resources may occasionally justify extended working hours, reliance on overtime as a routine strategy creates tensions between physician autonomy, non-maleficence, justice, and the sustainability of high-quality care [3,5].

From a bioethical perspective, protecting physicians promotes beneficence by supporting safe patient care, reinforces non-maleficence by reducing fatigue-related risks, advances justice by discouraging reliance on professional self-sacrifice to compensate for structural deficiencies, and respects physicians' autonomy by recognizing their legitimate right to preserve their own health and well-being.

Overtime should therefore not be considered merely an administrative challenge or a scheduling problem. It is a reflection of how healthcare systems distribute responsibility, occupational risk, and ethical obligations. A truly sustainable healthcare system must protect those who care for others, recognizing that physician well-being and patient safety are interconnected goals rather than competing priorities.

Addressing this issue requires moving beyond the concept of individual resilience. Although personal coping strategies have value, they cannot compensate for organizational models that consistently expose professionals to unsustainable demands. Effective responses require adequate workforce planning, realistic workload distribution, enforcement of working-hour policies, regular monitoring of working hours, appropriate rest and recovery periods, fatigue-risk management strategies, transparent overtime compensation, access to confidential mental health support, and leadership approaches that prioritize professional well-being and organizational accountability for maintaining safe working conditions [3,5].

Healthcare systems cannot remain sustainable if the health of those who sustain them is continually depleted. Protecting physician well-being is therefore not a concession to professionals but an ethical prerequisite for safe, equitable, and resilient healthcare.
